# Supplementary material for: A Qualitative Study to Understand the Impact of Caring for Traumatic Injury Survivors
Source: Int J Environ Res Public Health. 2022 Dec 3;19(23):16202. doi: 10.3390/ijerph192316202 (PMC9737134; doi:10.3390/ijerph192316202)
Supplement: Supplementary file 1 [file ijerph-19-16202-s001.zip › ijerph-1943946-supplementary.pdf]

Supplementary Material

Questionnaire S1. Demographic questionnaire

Carers of Traumatic Injury Survivors: Description of Injury and Carer Questionnaire.

Please complete the following questions.

What is today's date? | D | D | M | M | Y | Y |

1. What is your age? ____________________

2. Gender? ☐ Male ☐ Female

3. Can you briefly describe the injury of the person you care for?
   ____
   ____
   ____

4. How did their injury happen?

____
____
____

5. Where did their injury happen?
6.

____

7. How long is it since they had their injury?
   ☐ 0-6 months ☐ 6-12 months ☐ 12-18 months 18-[illegible]onths
   ☐ 2-3 years ☐ 3-4 years ☐ Over 4 years

8. Do you know what their injury severity score (ISS) score was when they had their injury?
   ☐ Yes, it was __________________ No, I can'[illegible]ember.

9. Do you know what their Glasgow Coma Scale (GCS) score was when they had their injury (if applicable)?
   ☐ Yes, it was ____________ No, [illegible]n't remember ☐ Not applicable.

10. How long did they stay in hospital after their injury? (Estimate days, weeks, months etc.)

11. Were you in paid employment, self-employed or doing voluntary work before their injury? If so, what work were you doing?

Approximately, how many hours were you working per week?

12. Are you still in paid employment, self-employed or doing voluntary work? If so, what work are you doing now?

Approximately, how many hours are you working per week?

Thank you for completing the questionnaire.

Topic Guide S2. Carer Topic guide

Questions	Prompts	Links to framework. ICF/ RAM modes
How has your employment been affected by caregiving?	• How has your employment situation changed since the time of the injury?<br>• If you are working, how have your relationships at work changed since becoming a carer?<br>• How would you describe the response of your employer to the occurrence of the traumatic injury?<br>• How does juggling your employment with being a carer or just being a carer affect you physically? E.g. in terms of energy levels.<br>• Has your enjoyment at work been affected by your joint role as a carer?	Functional<br>Social, Emotional<br>Emotional, Environmental<br>Physical, Functional<br>Personal factors, Environmental factors, Functional, Social.
What impact has the traumatic injury had on your household financially?	• How would you summarise the changes to your household financial situation since the time of the injury?<br>• How prepared were you for this at the time the injury took place?<br>- What information were you provided with about injury costs etc?<br>• Have you received financial support from anyone outside of your household since the injury?<br>- If yes, when was this and what was the nature of it?<br>• How have role changes within your household since the injury affected you financially?<br>• Have you experienced certain changes to your lifestyle resulting from the financial strain of the injury? E.g. no longer paying for a gym membership.	Personal factors<br>Environmental factors, Personal factors<br>Environmental factors, Emotional, Social<br>Functional<br>Environmental factors
How is your ability to work/financial status affecting your psychological wellbeing?	• How did adjusting to the new role as a carer affect your psychological wellbeing?<br>- Was this affected by your employment situation?<br>• Has uncertainty related to your financial /employment situation contributed to negative feelings?<br>• Do you experience stress as a result of the financial burden created by the injury?<br>- How would you describe the nature/ extent of this?<br>• Has social support from others mitigated the effect of any financial stress/ concerns you have experienced?<br>• How would you describe the effect that working outside (or inside) of the home has on your mood?	Functional, Emotional<br>Personal factors<br>Personal factors<br>Personal factors, Emotional<br>Environmental factors, Functional, Physical
Can you tell me about your social life and how	• How does your social life compare to what it was before the injury took place?	Personal factors, Social, Emotional

this is affected by being a carer?	• Are there any physical changes within yourself that affect this e.g. improved stamina /less energy?<br>• How would you say that performing caring activities impacts your ability to socialise outside the house?<br>• Are there any social relationships or activities you enjoy that you feel motivated to keep up?<br>• Have you developed new relationships /hobbies since becoming a carer?<br>- How has caring interacted with this?<br>• Are you ever encouraged to socialise outside the home by friends/ other members of your household?	Physical<br>Environmental factors, Functional, Social<br>Social, Emotional<br>Social, Emotional, Personal factors<br>Environmental factors, Emotional
To what extent have changes to your social life affected your psychological wellbeing?	• To what extent do you feel supported in your role by others, (non-injured people) and consequently able to socialise?<br>• Do you notice positive effects on how you feel as a result of socialising outside the home?<br>- How does this make you feel about being a carer?<br>• Do you ever feel concerned about your psychological wellbeing as a result of your levels of social engagement?<br>• Have you ever experienced negative feelings as a result of social participation?<br>• Do time limitations resulting from your role as a carer affect the stress associated with socialising?<br>• Are you able to be spontaneous in terms of your social participation?<br>- If yes/ no, how does this affect your general mood?	Personal factors, Emotional<br>Physical, Functional<br>Personal factors, Physical<br>Physical, Emotional, Social<br>Environmental factors, Functional, Physical<br>Social, Physical, Environmental factors

Table S1. Table of RAM modes with corresponding behaviours that influence adaptation to caring:

RAM mode/coping process:	Function/Stimuli in the environment responded to:
Physiological (physical)	Emphasises the person's physiological integrity e.g., maintaining physical stamina
Self-concept (emotional)	Deals with interpersonal relationships and social influences

Role function (functional)	Social integrity. Focuses on performing behaviours that are associated with the roles people carry out
Interdependence (social)	Emphasises behaviours related to developing and maintaining positive relationships
